# Metabolic engineering of *Oryza sativa* for lignin augmentation and structural simplification

**DOI:** 10.5511/plantbiotechnology.24.0131a

**Published:** 2024-06-25

**Authors:** Toshiaki Umezawa

**Affiliations:** 1 Research Institute for Sustainable Humanosphere, Kyoto University

**Keywords:** grass, lignin metabolic engineering, rice, structural simplification, upregulation

## Abstract

The sustainable production and utilization of lignocellulose biomass are indispensable for establishing sustainable societies. Trees and large-sized grasses are the major sources of lignocellulose biomass, while large-sized grasses greatly surpass trees in terms of lignocellulose biomass productivity. With an overall aim to improve lignocellulose usability, it is important to increase the lignin content and simplify lignin structures in biomass plants via lignin metabolic engineering. Rice (*Oryza sativa*) is not only a representative and important grass crop, but also is a model for large-sized grasses in biotechnology. This review outlines progress in lignin metabolic engineering in grasses, mainly rice, including characterization of the lignocellulose properties, the augmentation of lignin content and the simplification of lignin structures. These findings have broad applicability for the metabolic engineering of lignin in large-sized grass biomass plants.

## Introduction

In September 2015, the 2030 Agenda for Sustainable Development was adopted by the United Nations Sustainable Development Summit. The 2030 Agenda listed 17 Sustainable Development Goals to realize a sustainable world (https://www.mofa.go.jp/policy/oda/sdgs/index.html (Accessed Oct 16, 2023)). Then, in December of the same year, the Paris Agreement—a multilateral agreement on climate change control—was adopted at the 21st Session of the Conference of the Parties to the United Nations Framework Convention on Climate Change. It requires all participating countries to put forth their best efforts to reduce CO_2_ emissions, with the aim of achieving global warming countermeasures (https://unfccc.int/process-and-meetings/the-paris-agreement (Accessed Dec 29, 2023)). These frameworks are in line with the concept of a bioeconomy that aims to switch from fossil resources to sustainable biomass resources for various economic activities, by incorporating the idea of biotechnology in addition to global sustainability and renewability (Igarashi 2017). These activities are intrinsically consistent with the concept of the social common capital (Uzawa 2005).

Thus, the importance of renewable resources and energy as substitute for fossil energy and resources has increased over the past decade. In fact, the amount of electricity generated by solar and wind powers has increased substantially. However, their output fluctuates widely depending upon the weather conditions. In addition, electricity demand varies within a day, for example, between daytime and nighttime. The difference between the output fluctuations of solar and wind powers and the demand is compensated for by thermal power output adjustments. In this regard, the combustion of coal or other fossil fuels in thermal power plants must be reduced. The combustion of biomass as an alternative has recently attracted attention. Tree biomass combustion has increased by more than ten times during the last decade in Japan, and accounted for about 17.9% of total wood consumption in 2021 in Japan (https://www.rinya.maff.go.jp/j/kikaku/hakusyo/r4hakusyo/attach/pdf/index-6.pdf (Accessed Nov 30, 2023)). In addition, biomass combustion is still indispensable to meet rural and local energy demands in some countries. Moreover, among the renewable resources, only biomass resources can supply organic compounds such as industrial feedstock and liquid fuels, as a substitute, for example, for naphtha. Taken together, biomass resources are critical for establishing a sustainable society.

Among biomass resources, lignocellulose biomass accounts for the highest proportion of renewable terrestrial biomass accumulated on earth. It can be classified into two categories, tree lignocellulose and non-tree lignocellulose mainly derived from grasses. The worldwide annual consumption of tree lignocellulose biomass is estimated to be about 2 billion tons (Umezawa 2018). Trees are indispensable for the production of wood-based materials and paper, which accounted for half of the total tree lignocellulose biomass consumption, while the other half was burned as fuel in 2022 (Forestry Production and Trade, FAOSTAT, Food and Agriculture Organization of the United Nations, https://www.fao.org/faostat/en/#data (Accessed Dec 1, 2023)). On the other hand, the production of lignocellulose biomass derived from non-tree sources, mainly grass biomass, is estimated to be about 3.6 billion tons per year, which is used as a soil improver, animal feed, solid fuel and so force, but largely disposed of by incineration and landfill (Tye et al. 2016).

The amount of lignocellulose biomass produced by large-sized grass biomass plants is estimated to range from 7 to 93 ton ha^−1^ yr^−1^, which significantly exceeds that produced by trees (less than 20 ton ha^−1^ yr^−1^) (Umezawa 2018). In addition, lignin is generally easier to isolate from grasses than from trees (Umezawa 2018). Bioeconomic activities in the future will require large amounts of biomass for use in energy and material production. Therefore, high-productivity biomass, especially large-sized grass biomass will be critically important. At present, a significant part of the tree biomass used for fuel is obtained by natural forest logging (Umezawa 2018). Therefore, it is crucial to establish sustainable systems for the production and utilization of grass biomass (Umezawa et al. 2020).

Lignocellulose biomass comprises secondary cell wall of vascular plants and is mainly composed of polysaccharides (cellulose and hemicelluloses) and lignin. For the efficient use of polysaccharides during processes such as pulping, forage digestion, and enzymatic saccharification, lignin that encrusts them has been considered an obstacle. On the other hand, lignin is also an important and potential aromatic feedstock (Abu-Omar et al. 2021; Gao and Mortimer 2022; Pazhany and Henry 2019; Rinaldi et al. 2016; Wang et al. 2022). Moreover, lignin exhibits larger heating values than polysaccharides (Umezawa 2018; White 1987), and is, therefore, an important component when lignocellulose biomass is used for direct combustion. For example, lignin-derived substances in the waste liquor of pulp mills are valuable fuels that contribute significantly to the economics of pulp and paper industries. Therefore, lignin is important for the economy of the whole process of pulp and paper industries, even though lignin reduction may be beneficial for the pulping process (Umezawa 2018).

It has long been believed that the mitigation of the recalcitrance of lignin is critically important for the utilization of lignocellulose polysaccharides. To alleviate the recalcitrance, many studies have aimed to produce various transgenic plants with reduced lignin content (Halpin 2019; Mahon and Mansfield 2019; Pazhany and Henry 2019). In addition, lignin metabolic engineering focusing on introduction of easily-degradable structures into lignin macromolecules without reducing total lignin content has received more attention, because this can avoid the plant growth penalty (Ha et al. 2021) caused by reduced lignin content (Chandrakanth et al. 2023; Gao and Mortimer 2022; Halpin 2019; Lebedev and Shestibratov 2021; Mahon and Mansfield 2019; Mottiar et al. 2016; Ralph et al. 2019; Rinaldi et al. 2016).

As for primary utilization of lignin, its structural complexity is a bottleneck. In addition, the lignin content of grasses is generally lower than that of trees (Umezawa 2018). Therefore, structural simplification of lignin and augmentation of its content, especially in grasses, are important targets for lignin metabolic engineering (Umezawa 2018; Umezawa and Suzuki 2008; Umezawa et al. 2020). The structural modification of lignin may also be an effective way to increase the heating values of lignocellulose biomass, because *p*-hydroxyphenyl (H) lignin has a slightly larger heating value, followed by guaiacyl (G) lignin and syringyl (S) lignin (Takeda et al. 2019b; Umezawa 2018).

This review outlines the recent advances in research on lignin metabolic engineering, focusing on the augmentation of lignin content and simplification of lignin structures in grasses, mainly rice (*Oryza sativa*) as a model for large-sized grass biomass plants.

## Structure of grass lignin

**Figure 1:**
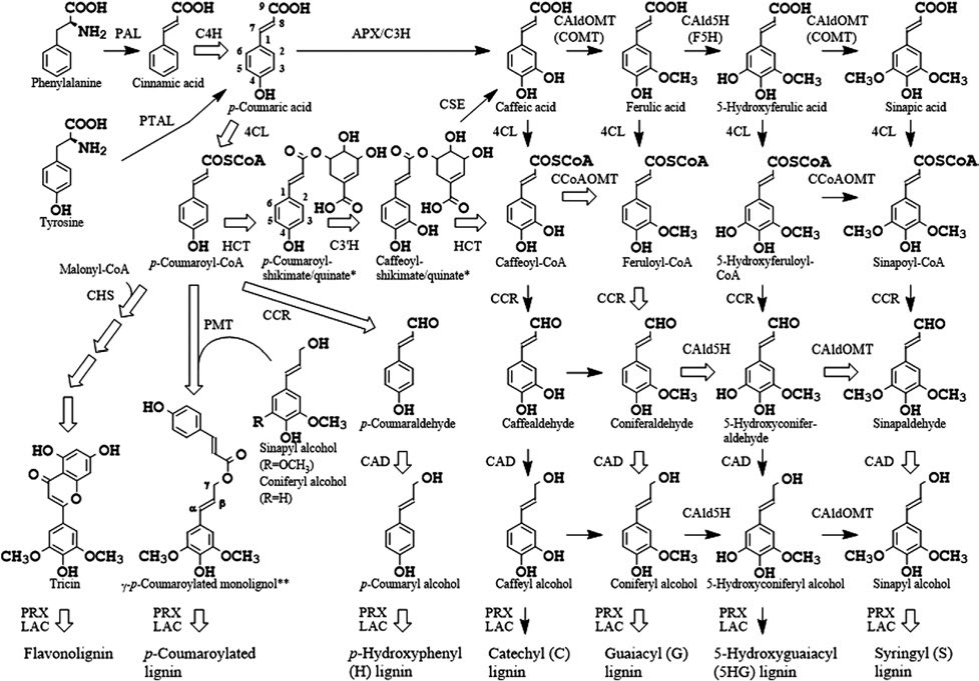
Figure 1. The cinnamate/monolignol pathway in grasses. Thick open arrow represents the major routes for lignin biosynthesis. C4H, cinnamate 4-hydroxylase; C3H, *p*-coumarate 3-hydroxylase; CAldOMT, 5-hydroxyconiferaldehyde *O*-methyltransferase; 4CL, 4-hydroxycinnamate:CoA ligase; HCT, hydroxycinnamoyl-CoA:shikimate/quinate hydroxycinnamoyltransferase; CCoAOMT, caffeoyl-CoA *O*-methyltransferase; CCR, cinnamoyl-CoA reductase; CAld5H, coniferaldehyde 5-hydroxylase; CAD, cinnamyl alcohol dehydrogenase; F5H, ferulate 5-hydroxylase; COMT, caffeic acid *O*-methyltransferase; C3′H, *p*-coumaroyl-shikimate/quinate 3-hydroxylase; PAL, phenylalanine ammonia-lyase; PTAL, phenylalanine/tyrosine ammonia-lyase; CSE, caffeoylshikimate esterase; PRX, peroxidase; LAC, laccase; CHS, chalcone synthase; PMT, *p*-coumaroyl-CoA:monolignol transferase; APX, ascorbate peroxidase. Only the structures of *shikimate ester and **γ-*p*-coumaroylated sinapyl alcohol are shown.

Lignin is a heterologous phenylpropanoid polymer that is synthesized through the coupling of phenoxyl radicals formed from three monolignols [*p*-coumaryl (4-coumaryl), coniferyl, and sinapyl alcohols] and related phenolic compounds including γ-acylated monolignols and tricin, a flavone, and so force (Figure 1) (Boerjan et al. 2003; Li et al. 2024; Ralph et al. 2019; Rinaldi et al. 2016; Vanholme et al. 2019). The aromatic compositions and chemical structures of lignin vary among the major phylogenetic divisions of the plant kingdom (i.e., gymnosperms, angiosperms, and grasses), but they are similar within each group, albeit with some variations. In addition, the amounts and aromatic composition of lignin also vary considerably among organs, cell types, developmental stages, and within cell wall layers. The content and structures of lignin are influenced not only by the genetic background of each species (Wahyuni et al. 2019; Widyajayantie et al. 2022), but also by environmental and nutritional conditions including biotic and abiotic stresses (Cesarino 2019; Rinaldi et al. 2016; Rivai et al. 2021).

Gymnosperm lignin is exclusively composed of G lignin with trace amounts of H lignin. Angiosperms produce both G and S lignin with small amounts of H lignin. Among angiosperms, grass family [Poaceae (Gramineae)] plants produce G and S lignin with slightly higher H lignin content than that of eudicots (Boerjan et al. 2003; Mansfield et al. 2012; Ralph et al. 2019; Vanholme et al. 2019), while grass lignin is significantly acylated, mainly at the γ-position of S lignin units, by *p*-coumaric acid and, to a lesser extent, by ferulic acid (Karlen et al. 2016, 2018; Ralph 2010). In addition, tricin is incorporated into lignin in grasses as a lignin monomer to produce another grass-characteristic lignin structure, flavonolignin or tricin-lignin units (del Río et al. 2012; Lam et al. 2023; Lam et al. 2021; Lan et al. 2015, 2016).

Evaluation of lignin content and structures is a fundamental task in lignin metabolic engineering. The metabolic engineering research activities produce a large number of transgenic plants and/or mutants to be analyzed, while the amounts of available plant samples are often very limited. Hence, high-throughput protocols for the lignin analyses are extremely valuable. The following high-throughput methods for the chemical degradation of lignin are available: thioacidolysis (Robinson and Mansfield 2009; Yamamura et al. 2012), nitrobenzene oxidation (Yamamura et al. 2010, 2021), and quantification of lignin (Hattori et al. 2012; Suzuki et al. 2009) and cell-wall bound *p*-hydroxycinnamates (Yamamura et al. 2011).

## Reduction of lignin content

In plant secondary cell walls, lignin co-exists with cellulose and hemicelluloses and encrusts cellulose microfibrils to form lignocellulose suprastructure. Consequently, lignin impedes the access of polysaccharide-hydrolyzing enzymes to cellulose and hemicelluloses, and there is generally a negative correlation between the lignin content and the enzymatic saccharification efficiency of lignocellulose materials. Over the last two decades, many studies have tried to improve the saccharification efficiency and forage digestibility of lignocellulosic biomass by reducing the lignin content using metabolic engineering methods, aiming at the social realization of the utilization of lignocellulosic polysaccharides (Barros et al. 2019b; Bhatia et al. 2017; Halpin 2019; Mahon and Mansfield 2019; Mottiar et al. 2016; Pazhany and Henry 2019; Rinaldi et al. 2016; Umezawa 2018; Wang et al. 2022).

Rice has been used as a model for large-sized grass biomass plants. Various transformants and mutants of rice (*O. sativa* L. ssp. *japonica* cv. Nipponbare) with lower lignin content and higher enzymatic saccharification efficiency compared with wild type control have been generated by knockdown or knockout of genes encoding enzymes in the cinnamate/monolignol pathway to produce lignin monomers (Umezawa 2010) (Figure 1) (Hattori et al. 2012; Koshiba et al. 2013a, 2013b). For example, RNA interference (RNAi)-induced rice transformants with downregulated expression of *O. sativa* *caffeic acid* O*-methyltransferase* (*COMT*, or *5-hydroxyconiferaldehyde* O-*methyltransferase*, *CAldOMT*) *1* (*OsCOMT1*) and the knockout mutants of the gene generated using the clustered regularly interspaced short palindromic repeats (CRISPR)/CRISPR-associated protein 9 (CRISPR/Cas9) technique exhibited lower content of lignin, especially S lignin, and higher enzymatic saccharification efficiency compared with wild type (Koshiba et al. 2013a; Martin et al. 2023). The culm of a *cinnamyl alcohol dehydrogenase2* (*cad2*) null mutant isolated from retrotransposon *Tos17-*insertion lines of rice (*O. sativa* L. ssp. *japonica* cv. Nipponbare) showed 16.1% higher enzymatic saccharification efficiency and 14.6% lower lignin content compared with those of the null segregant control (Koshiba et al. 2013b). The mutant exhibited brown-colored midribs in addition to hulls and internodes, clearly indicating both *brown midrib* (*bm*) and *gold hull and internode* (*gh*) phenotypes. This was the first example of a *bm* mutant in a C_3_ grass plant. The downregulation of *CAD* of another C_3_ grass model plant, *Brachypodium distachyon*, was achieved by artificial microRNA-mediated knockdown of *BdCAD1*, and this transformant also exhibited *bm* phenotype (Trabucco et al. 2013). Chemically induced *BdCAD1* mutants exhibited an intense reddish-brown coloration in the spikelets, flowers, rachilla, nodes, and lemma of the plant (d’Yvoire et al. 2013). The genes encoding COMT (CAldOMT) are also responsible for *bm* phenotype in some grasses such as maize (*Zea mays*) and sorghum (*Sorghum bicolor*) (Barrière et al. 2004; Sattler et al. 2010).

In the current social situation, it is timely to reassess the global feasibility of bioethanol production from lignocellulose biomass, including the technoeconomic analysis of the whole process from biomass cultivation to bioethanol production. In this regard, lignin metabolic engineering to reduce lignin content in sugarcane (Dias et al. 2009; Jung et al. 2012, 2016) may be a practical strategy to increase its saccharification efficiency, because sugarcane bagasse is consumed as fuel in sugar factories and its surplus can be exploited for saccharification followed by fermentation to produce additional bioethanol.

Meanwhile, these application-oriented studies aiming to reduce the lignin content and to exploit lignocellulosic polysaccharides have also provided new information about lignin biosynthetic mechanisms including the revision of the pathways to produce lignin monomers and the elucidation of the regulatory systems by transcription factors (Cesarino et al. 2016; Coomey et al. 2020; Deng and Lu 2017; Li et al. 2024; Miyamoto et al. 2020b; Nakano et al. 2015; Ohtani and Demura 2019; Rao and Dixon 2018; Xiao et al. 2021; Yao et al. 2021; Yoon et al. 2015; Zhang et al. 2020; Zhang et al. 2021; Zhong and Ye 2015). Moreover, these studies have provided new information about the assembly of lignocellulose components or supramolecular structures. For example, despite the general negative correlation between lignin content and enzymatic saccharification efficiency of lignocellulose materials, the parenchyma-rich inner part of the *Erianthus arundinaceus* internode did not show a negative correlation between the enzymatic saccharification efficiency and lignin content (Yamamura et al. 2013). This material showed distinct behavior in alkaline delignification from those of the outer part of the *Erianthus* internode as well as both parts of the sugarcane (*Saccharum* spp.) internode (Miyamoto et al. 2018). Studies of transgenic plants are expected to provide more direct information about lignocellulose supramolecular structures. Several studies have attempted to analyze the supramolecular structure of lignocellulose in lignin-related mutants and transformants of *Nicotiana tabacum* and Arabidopsis (*Arabidopsis thaliana*) (Carmona et al. 2015; Liu et al. 2016; Ruel et al. 2002, 2009). In rice, the above-mentioned *OsCAD2*- and *OsCAldOMT1* (*OsCOM1*)-deficient mutants and their double-knockout mutants were subjected to characterization of lignocellulose supramolecular structures (Martin et al. 2019, 2023). The disruption of both *OsCAldOMT1* and *OsCAD2* evidently affected the supramolecular structure, but the effects differed between the two genes. Compared with the deficiency of *OsCAD2*, that of *OsCAldOMT1* more prominently affected the lignocellulose supramolecular structures, resulting in higher cellulose mobility as primarily gauged by nuclear magnetic relaxation, at least for both mutants cultivated under employed conditions. These findings indicated that the two enzymes play differential roles in the formation of lignocellulose supramolecular structures (Martin et al. 2019, 2023). Another study showed that downregulation of *OsMYB103L* (synonym for *OsMYB103*) that can alter *OsCAD2* gene expression (Hirano et al. 2013) lead to changes in cellulose assembly (Wu et al. 2021).

The elucidation of lignocellulose supramolecular structures is an important goal in the field of cell wall science and lignocellulose science, both in terms of basic and applied science. Mutants in which the lignocellulose supramolecular structures are altered and/or the lignin content and structures are modified will play an important role in such research.

## Augmentation of lignin content

Breeding to augment the lignin content in biomass could improve its suitability as solid biomass fuel and increase the efficiency of the lignin biorefinery process (Scully et al. 2016; Umezawa 2013, 2018; Umezawa and Suzuki 2008; Umezawa et al. 2020). This is because lignin has larger heating values than polysaccharides and represents a potent source of valuable aromatic chemicals (Gao and Mortimer 2022; Mottiar et al. 2016; Pazhany and Henry 2019; Rinaldi et al. 2016; Umezawa 2018; White 1987). Two strategies for this purpose using rice (*O. sativa* L. ssp. *japonica* cv. Nipponbare) have been reported; the heterologous expression and endogenous overexpression of transcriptional activator genes (Koshiba et al. 2017; Umezawa 2013; Umezawa et al. 2020), and the knockout of endogenous transcriptional repressors (Miyamoto et al. 2019, 2020a; Umezawa et al. 2020).

Heterologous expression of three *A. thaliana* *MYB*s (*AtMYB55*, *AtMYB61*, and *AtMYB63*) in rice resulted in culms with increased lignin content (about 1.5-fold higher than that in control plants) (Koshiba et al. 2017). An in-depth lignin analysis suggested that heterologous expression of *AtMYB61* in rice increased the lignin content mainly by enriching S units as well as *p*-coumarate and tricin moieties in the lignin polymers, both of which are characteristic components of grass lignin (Koshiba et al. 2017). Similar results were reported for *Z. mays* MYB167. Heterologous expression of *ZmMYB167* in *Brachypodium* increased the lignin content (ca. 7% to 13%) and S lignin content (ca. 11% to 16%), and increased the content of cell wall-bound *p*-coumaric acid (ca. 15% to 24%) compared with controls, while its overexpression in maize produced transgenic plants with increased lignin (ca. 4% to 13%), *p*-coumaric acid (ca. 8% to 52%), and ferulic acid (ca. 13% to 38%) content (Bhatia et al. 2019). Similar studies on sorghum aiming to increase energy content in biomass plants were reported; the lignin content was augmented by overexpression of *SbMYB60* encoding a *S. bicolor* transcriptional activator in sorghum (Scully et al. 2016). Meanwhile, another study determined the lignin content of 30 Indonesian sorghum accessions, and identified those with high lignin content (Wahyuni et al. 2019). Recently, heterologous expression of *ZmMYB167* gene in *Miscanthus sinensis* was reported. Unlike the heterologous expression of *AtMYB61* in rice and of *ZmMYB167* in *Brachypodium* and the overexpression of *ZmMYB167* in maize, the heterologous expression of *ZmMYB167* in *Miscanthus* did not alter lignin composition or phenolic compounds, but increased the lignin content by ca. 15–24% compared with control plants, resulting in improved total energy levels of *Miscanthus* biomass, equivalent to 10% higher energy yield per hectare (Bhatia et al. 2023).

The knockout of endogenous transcriptional repressors also lead to increased lignin content. Rice mutants in which the transcriptional repressor *OsMYB108* gene was defected using CRISPR/Cas9-mediated genome editing exhibited increased total lignin content with preferential enrichment of the grass-characteristic γ-*p*-coumaroylated and flavonolignin units (Miyamoto et al. 2019). When other putative transcriptional repressor genes, *OsWRKY36* and *OsWRKY102*, were knocked out using CRISPR/Cas9-mediated genome editing (Miyamoto et al. 2020a), the single mutations of *OsWRKY36* and *OsWRKY102* significantly increased lignin content by up to 28% and 32%, respectively. The knockout effect was integrated in the *OsWRKY36*/*OsWRKY102*-double-mutant lines, showing even higher lignin content (by up to 41% compared with controls). Unlike the *OsMYB108* mutants, in depth lignin analyses showed that the relative abundance of guaiacyl units and *p*-coumarate residues in lignin were slightly higher and lower, respectively, in the WRKY mutants than in the wild-type lignin, revealing that the functions of OsWRKY36 and OsWRKY102 differ from that of OsMYB108 (Miyamoto et al. 2020a). These results strongly suggested that the WRKYs and their close homologs are promising breeding targets for improving the utilization properties of grass biomass using conventional screening of non-transgenic mutant lines as well as genome-editing-mediated mutation.

Furthermore, lignin plays significant roles in mitigating the effects of biotic and abiotic stresses (Cesarino 2019; Choi et al. 2023; Dabravolski and Isayenkov 2023; Dong et al. 2022; Miedes et al. 2014; Moura et al. 2010; Pratyusha and Sarada 2022; Wang et al. 2022; Yu et al. 2023b). It was also reported that lignin is the predominant cell wall factor that enhances lodging resistance in rice (Liu et al. 2018). In this regard, many studies have aimed to increase lignin content in rice by manipulating the expression of genes encoding regulatory proteins and enzymes and have reported mitigation of biotic and abiotic stresses.

For example, overexpression of *OsTF1L*, an *O. sativa* homeodomain-leucine zipper transcription factor gene, in rice promoted lignin biosynthesis and stomatal closure, which improved drought tolerance (Bang et al. 2019). Overexpression of *OsbHLH034*, an *O. sativa* jasmonate-responsive basic helix–loop–helix (bHLH)-type transcription factor gene, in rice induced bacterial blight resistance via an increase in lignin biosynthesis (Onohata and Gomi 2020). Overexpression of *OsNAC055* encoding an *O. sativa* transcription factor that directly activates the *O. sativa* lignin biosynthetic genes, *OsCCR10* and *OsCAD2*, increased the lignin content in rice straw. This transcription factor was found to regulate gibberellin-mediated lignin biosynthesis in rice straw (Liu et al. 2022). Ectopic expression of *OsDhn-Rab16D* (*OsjDHN5*), an *O. sativa* dehydrin gene, in rice increased lignin biosynthesis under drought stress (Tiwari et al. 2019). Heterologous expression of *SiMYB16*, a foxtail millet (*Setaria italica*) MYB-like transcription factor gene, in rice led to increased lignin content under a salt-stress treatment (Yu et al. 2023a). Transgenic rice plants overexpressing *OsNAC17* that promotes lignin accumulation in leaves and roots showed drought-tolerant phenotype compared with non-transgenic plants (Jung et al. 2022). *O. sativa* *cinnamoyl-CoA reductase 10* (*OsCCR10*) gene was found to be directly activated by OsNAC5 transcription factor, and the overexpression of *OsCCR10* in rice resulted in higher lignin content in roots and improved drought tolerance at the vegetative stages of growth compared with non-transgenic controls (Bang et al. 2022). Overexpression of *OsGRP3* encoding an *O. sativa* glycine-rich RNA-binding protein in rice enhanced lignin accumulation and drought tolerance (Xu et al. 2022). Knockout of *OsIDD2*, an *O. sativa* zinc finger and indeterminate domain (IDD) family transcription factor gene, produced rice mutants with slightly larger lignin content (Huang et al. 2018). Knockout of *OsMYB7* increased the lignin content in lamina joints of rice, and OsMYB7 was found to determine the leaf angle at the late developmental stage of lamina joints in rice (Kim et al. 2023). Some rice mutants also show elevated lignin content. Rice dwarf mutants with 2- to 3-fold increases in the content of total phenolic components including lignin in parenchyma cell walls of internodes were designated as *ectopic deposition of phenolic components1* (*edp1*), although the responsible gene has not yet been identified (Sato et al. 2011).

Grasses, especially rice, contain large amounts of silicon, which plays important roles in mitigating the effects of various biotic and abiotic stresses (Chack et al. 2023; Ma et al. 2011; Singh et al. 2021; Van Soest 2006). There is also a correlation between silicon content and lignin content in rice. Ma et al. identified silicon transporters, OsLsi1 and OsLsi2, in rice (Ma et al. 2006, 2007), and *OsLsi1*-deficient mutants exhibited higher lignin content and much lower silicon content in culms compared with wild type (Suzuki et al. 2012). In addition, wild-type rice grown under a hydroponic condition without silicon also exhibited a similar phenotype to the mutants, i.e., higher lignin content and very low silicon content compared with controls cultivated under conditions with sufficient silicon supply (Suzuki et al. 2012). Hydroponically grown sorghum with a limited supply of silicon also exhibited increased lignin content (Rivai et al. 2022). In addition, mature entire shoots of *B. distachyon* *low-silicon 1* (*Bdlsi1-1*) mutant also exhibited small but significant increases in lignin content compared with the wild-type control, although the lignin content in leaves, stems, spikelets, and entire shoots at the ripening growth stage was similar in the mutant and wild type (Głazowska et al. 2018).

Those experiments with mutants and hydroponically grown plants were probably well controlled and revealed the negative correlation between lignin content and silicon content. In contrast, experiments under biotic or abiotic stresses have yielded somewhat controversial results. There are reports of increased lignin content in rice amended with silicon under the biotic stress (the inoculation of a root-knot nematode *Meloidogyne graminicola*) (Zhan et al. 2018) and in wheat (*Triticum aestivum*) flag leaves supplied with silicon under the biotic stress (the inoculation of a pathogen that causes rice blast, *Pyricularia oryzae*) (Araújo et al. 2019). In addition, lignin content was increased in rice leaves inoculated with *P. oryzae*, and the increase was enhanced after silicon application (Ng et al. 2019). On the other hand, although increased lignin content of cell wall fraction in root-apex transition zone of rice was observed by the application of aluminium stress, the increase was suppressed by the addition of silicon (Jiang et al. 2022). These different results regarding changes in lignin content may be at least partly due to differences in cultivation and/or stress conditions and parts of the plant collected for analysis among different studies.

## Simplification of lignin structures

The complexity of lignin structures has long been a challenging bottleneck in its utilization as an industrial aromatic feedstock. In particular, lignin structures are more complex in grasses than in gymnosperms and eudicots (del Río et al. 2022; Ralph et al. 2019; Umezawa 2018). Therefore, the simplification of lignin structures is an important target of lignin metabolic engineering in grasses. Lignin structures can be simplified by controlling the lignin biosynthetic pathways to produce only H, G, or S lignin units (Gao and Mortimer 2022; Umezawa 2018; Umezawa et al. 2020). Such controls lead not only to simplification of the ratio of the units, but also to simplification of the population of lignin substructures namely intermonomer linkage structures. For example, S lignin unit inherently contains neither β-5, 5-5′, nor 4-*O*-5′ substructures and composed of more β-*O*-4 substructures, so it is simpler and more linear than G and H lignin units (Ralph et al. 2019; Umezawa 2018). Moreover, the simplification of lignin aromatic composition leads to a slight increase in its heating value. A higher number of methoxy groups in the lignin aromatic nuclei slightly reduces the heating value. Therefore, H lignin is estimated to have a larger heating values than G lignin, while S lignin has the lowest heating value (Takeda et al. 2019b; Umezawa 2018).

Gymnosperm lignin is composed exclusively of G lignin with trace amounts of H lignin, while eudicot lignin is composed of both G and S lignin with smaller amounts of H lignin. Like eudicot lignin, grass lignin also consists of G and S lignin but with slightly larger amounts of H lignin (Boerjan et al. 2003; Mansfield et al. 2012; Ralph et al. 2019; Vanholme et al. 2019). Furthermore, grass lignin is significantly acylated mainly by *p*-coumaric acid (Karlen et al. 2016, 2018; Ralph 2010), and contains flavonolignin or tricin-lignin units (del Río et al. 2012; Lam et al. 2023; Lam et al. 2021; Lan et al. 2015, 2016). Many metabolic engineering studies of eudicot lignin biosynthesis have been reported, and have produced various transgenic plants/mutants with elevated H, G, and S lignin content (Mahon and Mansfield 2019; Martarello et al. 2023; Pazhany and Henry 2019; Ralph et al. 2019; Rinaldi et al. 2016; Umezawa 2018; Wang et al. 2022). Some of them exclusively produced H (Bonawitz et al. 2014; Franke et al. 2002; Weng et al. 2010), G (Ciesielski et al. 2014; Meyer et al. 1998), and S lignin (Ciesielski et al. 2014) by knockout of the *C3′H* gene, knockout of the *CAld5H* gene, and upregulation of the *CAld5H* gene, respectively. However, most of those studies aimed to characterize gene functions and improve the usability of lignocellulose polysaccharides.

Although fewer studies have focused on grasses than on eudicots, there are a number that have successfully simplified the aromatic composition of grass lignin. To increase the S lignin content, rice transformants overexpressing *OsCAld5H1* (CYP84A5), encoding coniferaldehyde 5-hydroxylase (CAld5H;=ferulate 5-hydroxylase, F5H), which is located in the S lignin biosynthetic shunt in the monolignol biosynthetic pathway (Figure 1), were generated. The S units were enriched by 2.3-fold in the rice transformants compared with the control (Takeda et al. 2017). To increase the G lignin content, RNAi-mediated knockdown and CRISPR/Cas9-mediated loss-of function techniques were used to impair the function of *OsCAld5H1* in rice. Both the knockdown transformants and knockout mutants exhibited elevated G lignin content, with 1.2- and 1.5-fold enrichment of G lignin units compared with the control, respectively (Takeda et al. 2017, 2019a). Interestingly, however, the lignin in the *OsCAld5H1*-knockout mutans still contained considerable numbers of S units. In-depth lignin analyses revealed that the enrichment of G units in lignin of the mutants was limited to the non-γ-*p*-coumaroylated units, whereas the grass-characteristic γ-*p*-coumaroylated lignin units were almost unaffected. This result strongly suggested that CAld5H is mainly involved in the production of non-γ-*p*-coumaroylated S lignin units, common to both eudicots and grasses, but not in the production of the grass-characteristic γ-*p*-coumaroylated S units, at least in rice (Takeda et al. 2017, 2019a). In contrast, increased amounts of H lignin, lignin-associated ferulates, and tricin were detected in transgenic maize in which C3′H (ZmC3H1) was down-regulated using RNAi technology (Fornalé et al. 2015). The H lignin enriched rice plants were produced by RNAi- and CRISPR/Cas9-mediated techniques to impair the function of *OsC3′H1* (CYP98A4) (Takeda et al. 2018). The transcript level of *OsC3′H1* in the RNAi-mediated *OsC3′H1*-knockdown rice lines was about 0.5% of that in the control, and the transformants were able to reach maturity and set seeds, whereas CRISPR/Cas9-mediated *OsC3′H1*-knockout rice mutants were severely dwarfed and sterile. The lignin of mature *OsC3′H*-knockdown RNAi lines was largely enriched in H units (by 8-fold); the enrichment of H units was limited to non-acylated lignin units, with grass-specific γ-*p*-coumaroylated lignin units remaining apparently unchanged, similar to the case of the *OsCAld5H1* deficiency (Takeda et al. 2018).

These rice transgenic lines with distinct H/G/S aromatic unit ratios were used to study the impact of lignin composition on the chemical reactivity, enzymatic saccharification efficiency, and calorific value of rice lignocellulose (Takeda et al. 2019b). The H-lignin-enriched rice transgenic line showed significantly enhanced enzymatic saccharification efficiency after alkali and acid pretreatments, and even with no pretreatment. The S-lignin-enriched rice transgenic line displayed enhanced saccharification efficiency after a hot water pretreatment. However, although analyses of synthetic lignins (dehydrogenation polymer, DHP) comprising only H, G, or S units showed that H-DHP had highest heating value, followed by G-DHP and S-DHP in that order, the transgenic lines with higher proportions of H or G units did not show increased heating values. This may be ascribed at least in part, to incomplete simplification of lignin aromatic composition. These strategies to increase lignin content and to simplify lignin structures are also applicable to large-sized grass biomass plants, such as sorghum, switchgrass (*Panicum virgatum*), *Miscanthus* and *Erianthus*. When *S. bicolor* *F5H* (*SbF5H*) was overexpressed in sorghum, the transformant produced lignin with increased S lignin content and an increased ratio of S/G lignin, while plant growth and development remained relatively unaffected (Tetreault et al. 2020). Because the structures of grass lignin are more complex than those of gymnosperm and eudicot lignins, the thorough simplification of grass lignin structures is critical for the utilization of high biomass-producing large-sized grass biomass plants in the future. To achieve this goal, it will be important to elucidate the mechanisms of the formation of grass-characteristic lignin structures, such as *p*-coumaroylated lignin and flavonolignin units. In this regard, Lam et al. successfully eliminated the *p*-coumaroylated lignin unit in rice as described below (Lam et al. 2024).

As mentioned above, the γ-hydroxycinnamoylated S lignin units (mainly *p*-coumaroyl and feruloyl at a lesser extent) are characteristic of grass lignin structures (Karlen et al. 2016, 2018; Ralph 2010). The acylated units result from the coupling of radicals formed from γ-hydroxycinnamoylmonolignols and non-γ-acylated monolignols (Ralph 2010). In addition, hydroxycinnamates are found in grass cell walls at the C5 position of the arabinofuranosyl moiety of the xylan backbone. These esters are mainly ferulates, with a smaller proportion of *p*-coumarates (Buanafina 2009; Chandrakanth et al. 2023; Ralph et al. 2004). In relation to the formation of acylated lignin, the enzymes that acylate monolignols to produce γ-hydroxycinnamoylmonolignols, *p*-coumaroyl-CoA:monolignol transferase (PMT) and feruloyl-CoA:monolignol transferase (FMT) (Chandrakanth et al. 2023), have been identified in several grasses: rice (*O. sativa*), OsPMT1 (formerly OsPMT) (Lam et al. 2024; Smith et al. 2022; Withers et al. 2012), OsPMT2 (Lam et al. 2024), and OsFMT1 (Karlen et al. 2016; Smith et al. 2022); maize (*Z. mays*): ZmPMT (Marita et al. 2014) and ZmFMT (Smith et al. 2022); *Brachypodium* (*B. distachyon*): BdPMT1 (Karlen et al. 2016; Petrik et al. 2014) and BdPMT2 (Sibout et al. 2016); sorghum (*S. bicolor*): SbPMT (Smith et al. 2022) and SbFMT (Smith et al. 2022); and switchgrass (*P. virgatum*): PvPMT (Smith et al. 2022) and PvFMT (Smith et al. 2022). OsPMT1 and OsPMT2 function redundantly. In the *ospmt1 ospmt2* double-knockout mutant, *p*-coumarate units were undetectable in the lignin, and the lignin structure was successfully simplified by the elimination of *p*-coumaroyl decoration (Lam et al. 2024).

## Grass-characteristic lignin biosynthetic pathways

The formation of hydroxycinnamoylmonolignol is a step in a grass-characteristic pathway leading to hydroxycinnamoylated lignin units. Several studies over the last decade have provided new insight into the formation of the monolignols used in the biosynthesis of hydroxycinnamoylmonolignols. As mentioned above, although the *OsCAld5H1*-overexpression (Takeda et al. 2017), *OsCAld5H1*-knockdown/knockout (Takeda et al. 2017, 2019a), and *OsC3′H*-knockdown (Takeda et al. 2018) in rice efficiently modified the aromatic compositions to augment S, G, and H units, respectively, none of the transformants/mutants produced lignin composed of a single type of aromatic units. This contrasts sharply with the results obtained in Arabidopsis. For example, *A. thaliana* *CAld5H*-deficient *fah1* mutants produced lignins exclusively composed of G units (Ciesielski et al. 2014; Meyer et al. 1998), the *far1*-derived transformant in which *AtCAld5H* was overexpressed made lignins consisting only of S units (Ciesielski et al. 2014), and the *Arabidopsis C3′H-*deficient *ref8* mutant produced essentially only H units (Bonawitz et al. 2014; Franke et al. 2002; Weng et al. 2010). Interestingly, the in-depth lignin analysis showed that the modification of the abundance of the H, G and S units of the *OsCAld5H1*-deficient/overexpressed (Takeda et al. 2019a, 2017) and *OsC3′H*-deficient (Takeda et al. 2018) transgenic/mutant rice lines was because of changes in the content of non-γ-*p*-coumaroylated G and S units, whereas the content of γ-*p*-coumaroylated G and S units were largely unaffected. Moreover, in rice, the heterologous expression of *AtMYB61* (Koshiba et al. 2017) and knockout of *OsMYB108* (Miyamoto et al. 2019) resulted in the specific augmentation of γ-*p*-coumaroylated G and S units. Taken altogether, the results of those studies showed that rice, and possibly other grasses, has a parallel monolignol pathway to produce the grass-specific γ-*p*-coumaroylated monolignols; this pathway differs from the C3′H- and CAld5H-dependent conventional pathway that produces non-γ-*p*-coumaroylated monolignols (Umezawa et al. 2020). The mechanisms of the formation of monolignols used in the biosynthesis of γ-*p*-coumaroylated monolignols remain to be elucidated. In this regard, phenylalanine/tyrosine ammonia-lyase (PTAL) (Barros et al. 2016) and ascorbate peroxidase (APX/C3H), which has C3H activity to hydroxylate *p*-coumaric acid to afford caffeic acid (Barros et al. 2019a), are likely to be at least partly responsible for the formation of γ-*p*-coumaroylated monolignols (Chandrakanth et al. 2023). In addition, it was recently reported that two *O. sativa* 4-coumrate:CoA ligases, Os4CL3 and Os4CL4, play differential roles in the biosynthesis of non-γ-*p*-coumaroylated monolignols and γ-*p*-coumaroylated monolignols, providing further support for the existence of dual pathways (Afifi et al. 2022).

Studies during the last decade have shown that the flavone tricin is incorporated into lignin in grasses as a lignin monomer to produce a grass-characteristic lignin structures, namely tricin-lignin or flavonolignin units (del Río et al. 2012; Lam et al. 2023; Lam et al. 2021, 2022; Lan et al. 2015, 2016). In this regard, the OsROMT9 (=OsCOMT1 and OsCAldOMT1)-knockout rice mutant showed a 46% reduction in soluble tricin accumulation compared with wild type, which strongly suggested that OsROMT9 is the major OMT involved in tricin biosynthesis (Lam et al. 2015). This was in line with the reduced levels of the tricin-lignin (flavonolignin) units in a maize mutant (*brown midrib3*, *bm3*) of *ZmOMT*, a maize homolog of *OsCOMT1* (Fornalé et al. 2017), and in a sorghum mutant (*brown midrib12*, *bmr12*) of *SbOMT*, a sorghum homolog of *OsCOMT1* (Eudes et al. 2017). Biochemical analyses confirmed the dual function of OsCAldOMT1 (OsROMT9); one is the role as a key enzyme to produce sinapyl alcohol in S lignin synthesis, and the other is the dual methylation of flavone precursors to produce tricin in rice (Lam et al. 2023; Lam et al. 2019, 2021; Umezawa et al. 2020).

## Concluding remarks

Large-sized grasses are the important sources of lignocellulose biomass for the establishment of sustainable society because of their very high biomass productivity. However, the structures of grass lignin are more complex than those of gymnosperm and angiosperm lignins. Furthermore, in general, the lignin content is slightly lower in grasses than in gymnosperm and angiosperm trees. Hence, the structural simplification and augmentation of lignin content in grasses are important targets for grass lignin metabolic engineering. Recent research in this field has provided the basic knowledge about how to increase the lignin content and simplify its structure. These studies also revealed the presence of a new grass pathway or pathways leading to the grass-characteristic lignin structures, although final conclusions await further studies. Furthermore, research on lignin structures and biosynthesis in grasses has made substantial contribution to related scientific fields such as crop breeding, plant pathology, plant nutrition, and soil science, as well as plant physiology.

## Abbreviations

4CL: 4-hydroxycinnamate:CoA ligase
APX: ascorbate peroxidase
bHLH: basic helix–loop–helix
*bm*: *brown midrib*
*bmr*: *brown midrib* of sorghum
C3H: *p*-coumarate 3-hydroxylase
C3′H: *p*-coumaroyl-shikimate/quinate 3-hydroxylase
C4H: cinnamate 4-hydroxylase
CAD: cinnamyl alcohol dehydrogenase
CAld5H: coniferaldehyde 5-hydroxylase
CAldOMT: 5-hydroxyconiferaldehyde *O*-methyltransferase
Cas9: CRISPR-associated protein 9
CCoAOMT: caffeoyl-CoA *O*-methyltransferase
CCR: cinnamoyl-CoA reductase
CHS: chalcone synthase
COMT: caffeic acid *O*-methyltransferase
CRISPR: clustered regularly interspaced short palindromic repeats
CSE: caffeoylshikimate esterase
DHN5 (Dhn-Rab16D): YSK2-type dehydrin protein
DHP: dehydrogenation polymer (synthetic lignin)
*edp*: *ectopic deposition of phenolic components*
F5H: ferulate 5-hydroxylase
*fah*: *ferulic acid 5-hydroxylase*
FMT: feruloyl-CoA:monolignol transferase
G lignin: guaiacyl lignin
*gh*: *gold hull and internode*
GRP: glycine-rich RNA-binding protein
H lignin: *p*-hydroxyphenyl lignin
HCT: hydroxycinnamoyl-CoA:shikimate/quinate hydroxycinnamoyltransferase
IDD: indeterminate domain
LAC: laccase
*lsi*: *low silicon*
MYB: myeloblastosis
NAC: NAM, ATAF1,2, CUC2
PAL: phenylalanine ammonia-lyase
PMT: *p*-coumaroyl-CoA:monolignol transferase
PRX: peroxidase
PTAL: phenylalanine/tyrosine ammonia-lyase
*ref*: *reduced epidermal fluorescence*
RNAi: RNA interference
S lignin: syringyl lignin
*TF*: *transcription factor*
